# Artificial lightweight aggregate made from alternative and waste raw materials, hardened using the hybrid method

**DOI:** 10.1038/s41598-024-67454-3

**Published:** 2024-07-23

**Authors:** Agata Stempkowska, Tomasz Gawenda

**Affiliations:** Faculty of Civil Engineering and Resource Management, AGH University of Krakow, Mickiewicza 30 Av., 30-059 Krakow, Poland

**Keywords:** Artificial lightweight aggregate, Green roofs, Waste materials recycling, Water retention capacity, Civil engineering, Environmental sciences, Materials science

## Abstract

Lightweight aggregates are a material used in many industries. A huge amount of this material is used in construction and architecture. For the most part, lightweight construction aggregates are obtained from natural resources such as clay raw materials that have the ability to swell at high temperatures. Resources of these clays are limited and not available everywhere. Therefore, opportunities are being sought to produce lightweight artificial aggregates that have interesting performance characteristics due to their properties. For example, special preparation techniques can reduce or increase the water absorption of such an aggregate depending on the needs and application. The production of artificial lightweight aggregate using various types of waste materials is environmentally friendly as it reduces the depletion of natural resources. Therefore, this article proposes a method of obtaining artificial lightweight aggregate consolidated using two methods: drum and dynamic granulation. Hardening was achieved using combined methods: sintering and hydration, trying to maintain the highest possible porosity. Waste materials were used, such as dust from construction rubble and residues from the processing of PET bottles, as well as clay from the Bełchatów mine as a raw material accompanying the lignite overburden. High open porosity of the aggregates was achieved, above 30%, low apparent density of 1.23 g/cm^3^, low leachability of approximately 250 µS. The produced lightweight aggregates could ultimately be used in green roofs.

## Introduction

Lightweight aggregate (LWA) is defined as solid substance with an apparent density of less than 2.0 g/cm^3^ and a bulk density of less than 1.2 g/cm^3^^1^. LWAs are porous and often granular materials that are widely used in architecture, landscaping and geotechnics. The porosity of these materials can be either open or closed, which affects their applicability. For example, aggregates with open porosity can provide effective drainage but also micro water retention. Furthermore, they can provide better sound absorption and thermal insulation. Lightweight aggregates can be divided into the following categories^2–4^:

1. Naturally occurring raw materials that require further processing, such as clays and intrusive shales and vermiculite;
2. Naturally occurring raw materials that do not require processing, such as pumice stone, foamed lava, volcanic tuff and porous limestone.
3. Industrial wastes such as sintered fly ash or foamed blast furnace slag.

The most commonly used artificial aggregate in many industries is sintered fly ash. The use of lightweight aggregate in concrete has gained popularity due to its low density, good thermal conductivity and strength, environmental friendliness and many other advantages^5,6^.

A global environmental problem due to the use of large amounts of natural resources is generated by the construction industry. Most of the aggregates used in this industry are obtained from natural resources. There is a continuous increase in the production of construction materials and therefore non-renewable natural resources are diminishing at an accelerating rate due to the high demand for their use in concrete production. Of course, for heavy-duty, high-strength and cover concretes, the use of quality natural aggregates appears to be essential. However, modern construction is moving away from oversized heavy structures. The development of the manufacture of artificial of lightweight materials such as lightweight aggregate will help minimize the use of natural resources. Lightweight aggregate is significantly different from conventional aggregate. The obtained modifications may bring benefits and new challenges for designers for many reasons, for example weight reduction, improved acoustic or thermal properties, drainage or filtration capabilities^2,4,7^.

Undoubtedly, the consumption of construction aggregates depletes natural resources and poses a direct threat to the environment. Due to the increasing expansion of construction, resources of natural aggregates are rapidly decreasing. There is a local shortage of resources that requires proper use for sustainable development^8–10^. Bibliographic research conducted by the authors shows that the dominant issue in the field of waste and recycled materials is the technology of building materials, and in particular the technology of concrete. It can be said that these issues are present in virtually every scientific and economic field. Starting from mineral and biotic resources, through broadly understood chemical and physical sciences, to geotechnics^11^. An interesting direction of use of waste materials are fired materials such as bricks, tiles, lightweight aggregates^12–15^.

However, the authors of the above publications point out the difficulties in using waste materials. Despite homogenization processes, mineral waste is characterized by low composition stability and the presence of soluble compounds. Such compounds may cause efflorescence on the surface or increased leachability of harmful substances, which causes functional defects. Natural sand can be replaced using by-products from coarse aggregate production, which are widely available, cheaper and reduce the extraction of natural sand^16^. Additionally, manufactured sand has become a popular choice to replace natural sands because it is often extracted from streambeds and sand extraction is considered environmentally harmful^3^. In addition, the produced sand, which is made from hard igneous, sedimentary or metamorphic rocks, can be produced locally, reducing transportation costs.

The demand for aggregates with a coarser fraction is different. Such materials, obtained from waste raw materials, must be integrated. They can be obtained in several ways, such as:

- cold consolidation ~ 25 °C (cold setting, cementation, geopolymerization)^17,18^
- autoclaving to about 200 °C
- sintering, at temperatures above 900 °C^7^.

Generally, lightweight aggregate produced at, for example, ~ 1200°C provides the best mechanical properties, but deteriorates its porosity. To obtain the most favorable properties of artificial aggregate, the sintering temperature should be determined individually depending on the raw materials used, using available techniques, e.g. high-temperature microscopy (HSM). The sintering method also enables the production of aggregate in a short time. However, sintering requires a large amount of energy during production, which affects its price. Generally, the sintering method is widely used around the world in some popular commercial products such as LECA and Lytag. These are some of the most popular artificial lightweight aggregates that have been commercialized on the market to replace natural aggregate. Due to costs, energy consumption and CO_2_ emissions, new methods such as cold bonding and autoclaving are being investigated.

In the autoclaving method, the hardening and strength of the granules are achieved by pressure and temperature. There are research papers describing the autoclaving process for the production of aggregates^19,20^. In their research, the authors dry the aggregate at room temperature for 24 h and then harden it at a temperature of up to 200° C for several hours. Through this procedure, they obtained aggregate very quickly. Tests have shown that a small amount of binding material is required to consolidate the aggregate. Unfortunately, there is not enough research on the effectiveness of the autoclaving method. This is because this method requires a specialized machine with precise temperature and pressure control to harden the aggregate. Furthermore, the autoclave is very expensive and requires high energy consumption and large production facilities to complete the process.

The third popular bonding method is cold bonding. It is a process of strengthening larger particles obtained using pressure or pressureless agglomeration methods. In the cold setting process, cement or an alkaline activator is usually used as a binder^18,21,22^. The authors indicate that the cold bonding method was considered profitable because consolidation takes place at room temperature. Compared to other production processes, the cold bonding method minimizes energy consumption. In the case of cold gluing, the granules are dried at room temperature for at least 24 h. Then, such granules require hardening, preferably in a closed chamber with steam, until the required strength is achieved^17,20,23^. The main challenge with cold set aggregates is the requirement for longer production times, as curing is typically required for 28 days. It is not always reasonable or possible to use cements or other alkaline activators.

Regardless of the uses of artificial aggregates in concrete production, LWA is worth investigating in particular to minimize environmental problems, along with maintaining long-term sustainability through improving water quality (filtration)^24^ or as a substrate for green roofs to mitigate the urban heat island effect^25,26^. As a sustainable ecosystem system, the green roof is known for its ability to provide thermal resilience and buffer surface runoff of stormwater in urban areas. The shape and type of materials used in the drainage of the green roof and the substrate layer significantly affect the energy efficiency and water drainage^27–31^. Due to the larger number of internal contact pores in the lightweight aggregate, moisture absorption is faster than with regular aggregate.

Ecological issues, such as the limitation of natural resources and huge amounts of waste, are increasingly leading the developing civilization towards sustainable construction. The two main environmental problems are the depletion of natural resources and the disposal of waste generated during various processes.

Therefore, the authors attempted to produce a new lightweight aggregate from by-products and waste materials^32^. In the presented research works, the authors used a combined (hybrid) method—sintering and hydration hardening. Another objective is to obtain an aggregate with an appropriately shaped open porosity, one that provides as much water retention as possible.

## Materials and methods

### Characteristics of the analytical methods

In order to assess the mineral content of individual samples, X-ray analysis was performed. The tests were performed using a PANanalytical X-ray diffractometer model (Empyrean, Malvern Eindhoven, Netherlands). The share of individual phases was determined using the Rietveld method. The measurements were made using monochromatic radiation with a wavelength corresponding to the Kα1 emission line of copper in the angle range 5–90° on the 2ϴ scale. The qualitative analysis of the phase composition was carried out using the X ‘Pert HighScore plus 3.0 Plus computer program developed by PANanalytical. The reference databases used were PDF-2 (2004) and ICSD Database FIZ Karlsruhe (2012). In order to determine the elemental composition of the samples, the following was used: X-ray fluorescence method XRF (Rigaku—Primini WDXRF spectrometer Tokyo, Japan).

Measurements in the high-temperature microscope (HSM, Misura® Expert System Solution, Modena Italy) were performed on particular sets of specimens with the temperature increment 10 °C/min Cylindrical test samples (ø = 2 mm, h = 3 mm) were pressed manually. This allows the influence of temperature on the behavior of the material under investigation to be evaluated. The greatest advantage of this method is the ability to make in situ observations of changes in the dimensions and shape of the sample as it is heated.

The samples were fired in an FCF 12SHM electric furnace from Czylok, Poland, operating temperature up to 1250 °C, working chamber with a capacity of 8 dm^3^.

Differential thermal analysis concrete plastics and gas emissions were determined using the STA 449 F3 Jupiter Thermal Analyzer (Netzsch, Germany) and the coupled quadrupole mass spectrometer TA-QMS Coupling (Netzsch, Germany). The measurement was carried out in alumina crucibles at a heating rate of 10 °C/min in a temperature range 30–600 °C in an air and argon atmosphere with a constant flow of 20 ml/min.

In order to determine the basic mechanical properties, an abrasion testing device was used. The tests were carried out on an apparatus from Erweka TAR II, (Frankfurt, Germany). The result of the test is the percentage weight loss of the sample with a grain size of less than 2.0 mm. The test time was 10 min, with a tank speed of 20 rpm. The hardness test was carried out using an ERWEKA TBH 125 TD (Frankfurt, Germany). The measuring range of the hardness station is 10–500 N, minimum grain diameter 2.0mm. Measurements were taken at a constant contact pressure.

The microstructure of the tested materials was examined using a scanning microscope (HITACHI HITACHI S-4700 with microanalysis system NORAN Vantage, Japan) This microscope allows operation under high vacuum and operation with steam as the working gas in low vacuum mode (10–200 Pa). Achievable magnifications range from 100 × to 500 000 × . The microscope makes it possible to assess the surface of materials, internal structure, changes and deformations.

### Characteristics of the raw materials used

#### Concrete recycled dust

**Figure 1:**
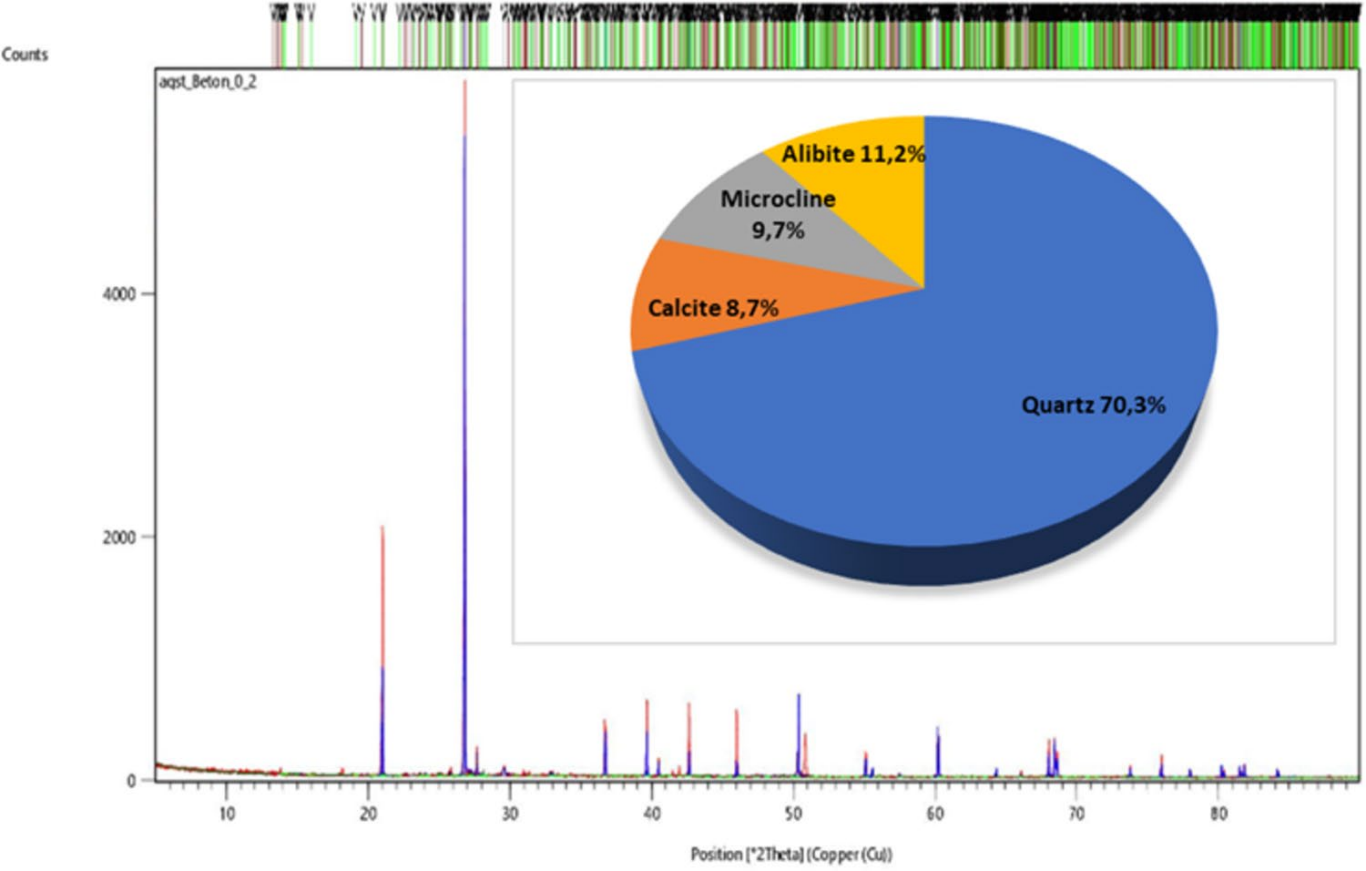
X-ray image with quantitative analysis of concrete dust from demolition.

Construction waste from construction sites of new buildings, demolitions, modernization of existing buildings or from road infrastructure constitutes approximately 32% of the total mass of waste in the world. Among them, the largest share is construction rubble: concrete and brick^33–35^. Concrete dust (0-2mm), generated during the production of coarse aggregates, is also produced from recycling elements from large slab blocks. These are prefabricated elements of various shapes and dimensions made of reinforced concrete elements that make up the structure of the block walls. They were widely used during communism in Poland and the GDR because they had a short construction time, but their durability was not ensured at a high level, which is why today there are a large number of prefabricated elements from demolitions or demolitions. The current situation in Ukraine also causes the formation of large amounts of large slab debris. Currently, concretes are constantly being improved and numerous studies are being carried out on the introduction of waste materials into their structure^36^. Concrete dust was used for two reasons. Firstly, we wanted to achieve less plasticity in the mass, and secondly, we hypothesised that the binding properties of the recovered cement would regenerate after firing. Figure 1 shows the diffractogram of concrete dust used in the tests. The main crystalline phases are silica SiO_2_ in the amount of 70.3%wt., calcium carbonate CaCO_3_ in the amount of 8.7%wt (calcite, watertite) and aluminosilicates of sodium Na[AlSi_3_O_8_] (albite) 11.2%wt and potassium K[AlSi_3_O_8_] (microcline) 9.7%wt.

#### Waste clay from the Bełchatów open pit mine

Coal clay from Bełchatów was used for plasticization and as a binder. Plasticization is the ability to create a mass using at least two products (water-sensitive, i.e. plastic raw material, e.g. clay, and water), which can be shaped in a selected way when wet, and retains its form after drying. Drying causes a loss of plasticity, but it is regained upon repeated contact with water; to obtain a permanent loss of plasticity, a firing process is used. Laboratory tests of the composition and physicochemical properties of samples of overburden rocks in deposits, i.e. boulder clays, silts and clays, in terms of their potential use, have been carried out at the Bełchatów Mine for many years, and the best of them are selectively extracted to mineral dumps. Rocks rich in smectites, which include the tested clays, could be useful for various applications^34,35,37,38^. However, no work has been undertaken so far on using them for lightweight aggregates. The current resources in the deposit can be extracted in large, even industrial quantities. The average chemical composition of clay samples is given in Table 1. They are characterized by a relatively high Al_2_O_3_ content of 24.06% by weight for clay rocks. The presence of Fe_2_O_3_ 3.87% wt. is quite clearly visible. In turn, the following can be considered low: the amounts of alkalis Na_2_O and K_2_O in total do not exceed 1% by weight, the CaO content is 1.55% wt. and organic parts 0.5%wt. There are also traces of sulphur content (given in the oxide form)—0.1% wt. The total loss on ignition was estimated at 4.46%wt and the average moisture was 8.92%wt.

**Table 1:** Oxide chemical composition of the clay used to produce the aggregate.

Element %wt	SiO_2_	Al_2_O_3_	Fe_2_O_3_	TiO_2_	CaO	MgO	MnO
	63.67	24.06	3.87	0.43	1.55	1.1	0.02
	K_2_O	Na_2_O	SO_3_	P_2_O_5_	Water	LOI	Organic
	0.4	0.08	0.1	0.05	8.92	4.46	0.5

#### Residues from processing PET bottles

Finding a use and then reusing PET bottles is a very good ecological step, because this product is used on a huge scale all over the world as packaging for various types of drinks and water. Due to their properties, PET packaging decomposes slowly, taking up to several hundred years, so it is important to reuse them. However, over time, through repeated use of the same waste, the material loses its properties and continuous production of bottles is impossible. Therefore, it is important to constantly look for opportunities where each type of waste could be successfully used. The material used in the research is final waste from the recycling process containing PET dust, label remnants (paper, foil) and other post-process residues. Table 2 shows the chemical composition of PET waste used in the research, obtained using the XRF method. The main ingredients identified were silicon, calcium and iron oxides. No oxides of harmful and toxic elements such as mercury, cadmium or lead were detected. However, this method has limitations in the identification of light elements such as H, O, C. It is these elements that polyethylene terephthalate C_10_H_8_O_4_ consists of. The average ash content was determined to be 15.91%wt the remaining compounds burn out.

**Table 2:** Oxide composition of PET waste after calcination.

Element %wt	MgO	Al_2_O_3_	SiO_2_	P_2_O_5_	SO_3_	Na_2_O	K_2_O
	2.73	5.79	32.92	0.76	0.70	0.34	1.44
	CaO	TiO_2_	MnO	Fe_2_O_3_	CuO	ZnO	SrO
	45.77	1.17	0.49	6.61	0.32	0.60	0.23

**Figure 2:**
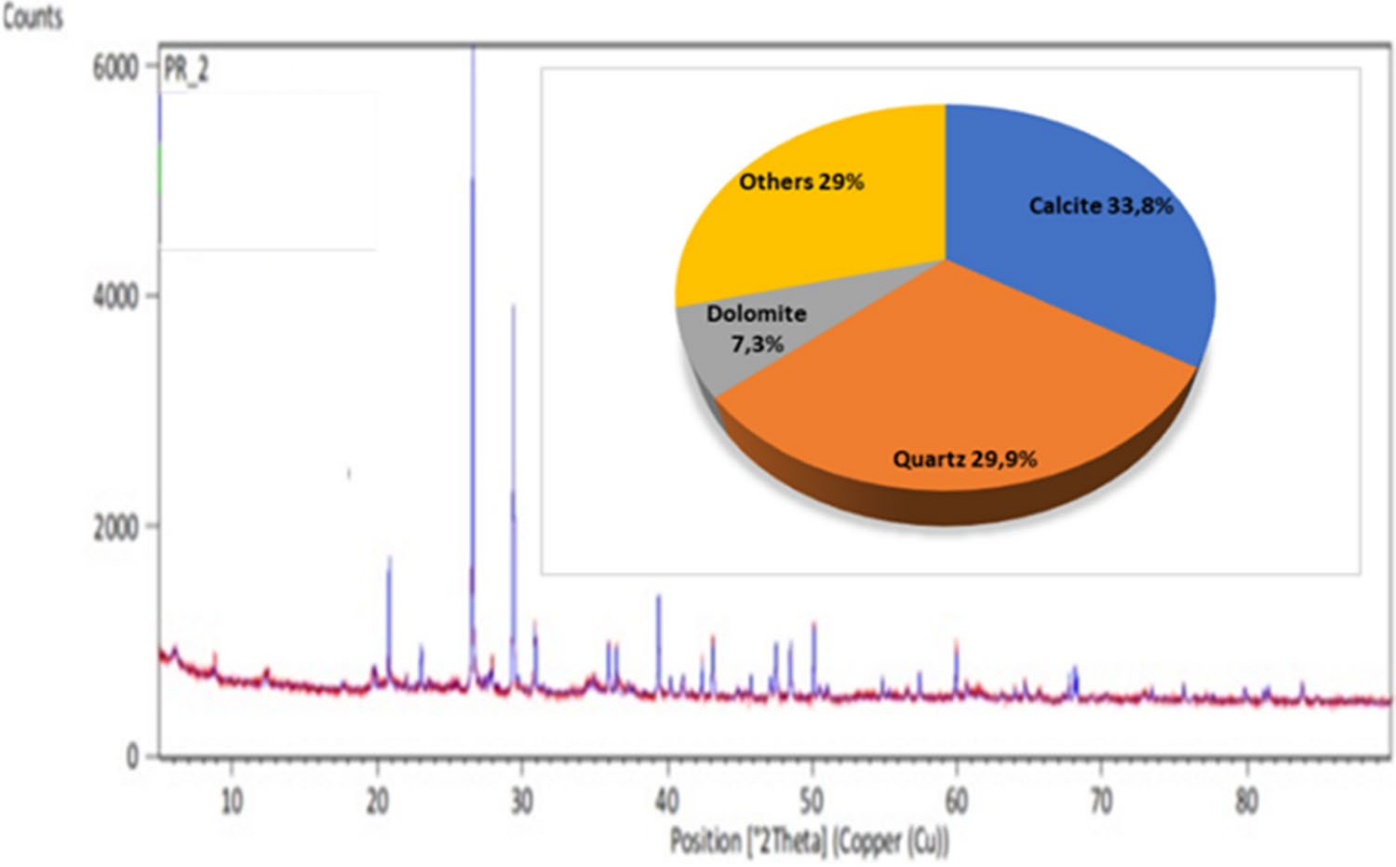
X-ray of PET waste with estimated quantitative analysis.

The calorific value of the waste was also tested—this is to improve the efficiency of sintering. The heat of combustion in the dry state Qs amounted to 29,590 kJ/kg on average, and the emission of chlorine to the atmosphere was 0.80%wt. The calorific value of waste, PET, generates an exothermic reaction through combustion, which reduces energy consumption in the sintering process. This contributes to savings in the energy demand of the furnace, which results in lower gas emissions into the environment and, therefore, economic savings. The total moisture of the waste was 15.6% by weight. The analysis of the phase composition of the tested waste was very difficult. Only three phases that may be included in the tested material were identified. These are calcite, silica and dolomite (Fig. 2). The authors decided to provide the quantitative composition of the identified minerals, but it should be taken into account that this is an estimated analysis.

**Figure 3:**
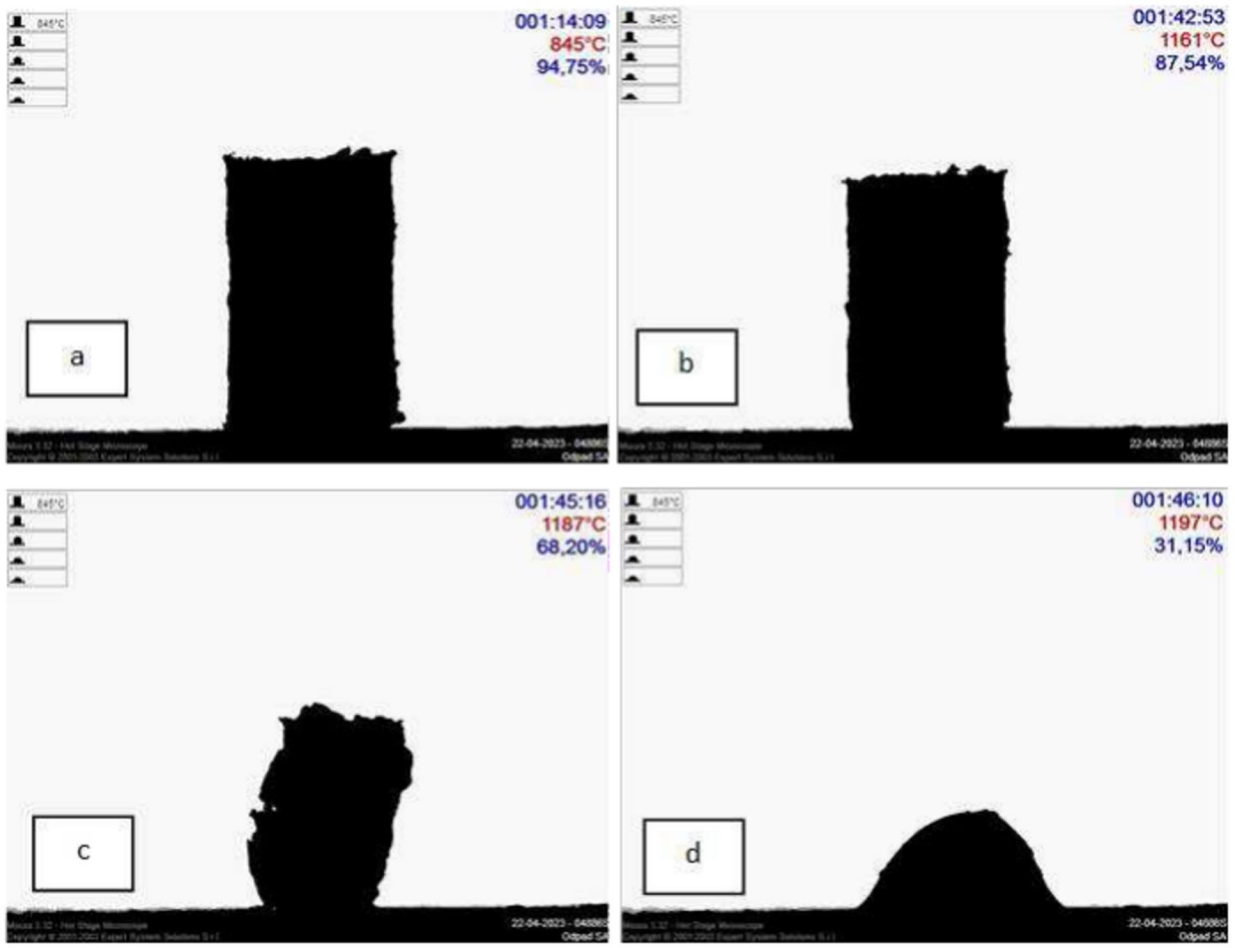
Images of PET waste obtained from a high-temperature microscope, initial stage—5% change in dimensions (**a**), beginning of a rapid geometric change (**b**), combustion of the PET material (**c**) loose ash (**d**).

Since the ultimate method of aggregate consolidation is sintering, tests using high-temperature microscopy (HSM) were carried out to illustrate changes in the sample geometry. Figure 3 shows the research results. In the case of testing PET waste, no temperatures characteristic of sintered materials were observed in the sample. A geometric change called "corner rounding" was recorded at 845°C (5% change in sample dimensions) (a). Then the material shrinks and decomposes more and more without sintering. From a temperature of 1160°C, a rapid change in the dimensions of the sample is observed (b,c). At a temperature of 1200°C, only loose ash remains (d), consisting mainly of silica and calcium oxide.

### Manufacturing method – granulation

**Figure 4:**
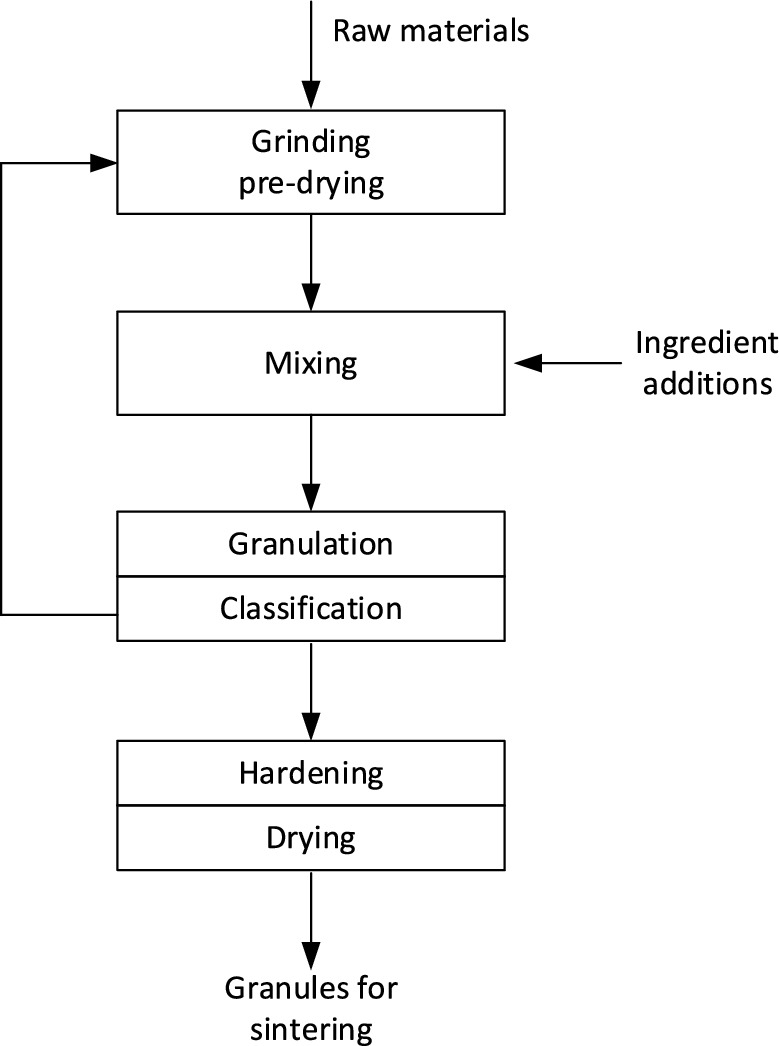
Block diagram of lightweight aggregate production.

The production process for artificial aggregate consists of five stages. In the first stage, the raw materials are dried. Drying is very important so that the resulting material is homogeneous and does not clog the mill's working spaces. In this research, we used dry grinding and homogenisation technologies. The concrete dust and PET paste did not need to be pre-ground. Clay was pre-ground because we were concerned that this material would not homogenise sufficiently. Ball mills were used in the process. In the second stage, the appropriately selected ingredients are mixed until the mixture reaches optimum homogenisation, the homogenisation time was 20 min. In the third stage, the raw material mixture is subjected to a granulation process by agglomeration of fine particles using water. In the fourth stage, the material is dispersed and the classification of the granules according to grain size takes place, and the fine products and damaged granules are returned to the granulating process circuit. The hardening of the fresh granules in the fifth stage is achieved by means of drying. This is followed by the granule firing process, which is discussed later in this article. The flowchart of lightweight aggregate production can be illustrated in Fig. 4. The consolidation process, called structured agglomeration of powders with a high degree of dispersion, aims to combine small dust particles to create larger aggregates (over 1 mm in size) with appropriate properties. This process is used to obtain a form of product that will be convenient for use by recipients or possible for further use in appropriate technologies.

**Figure 5:**
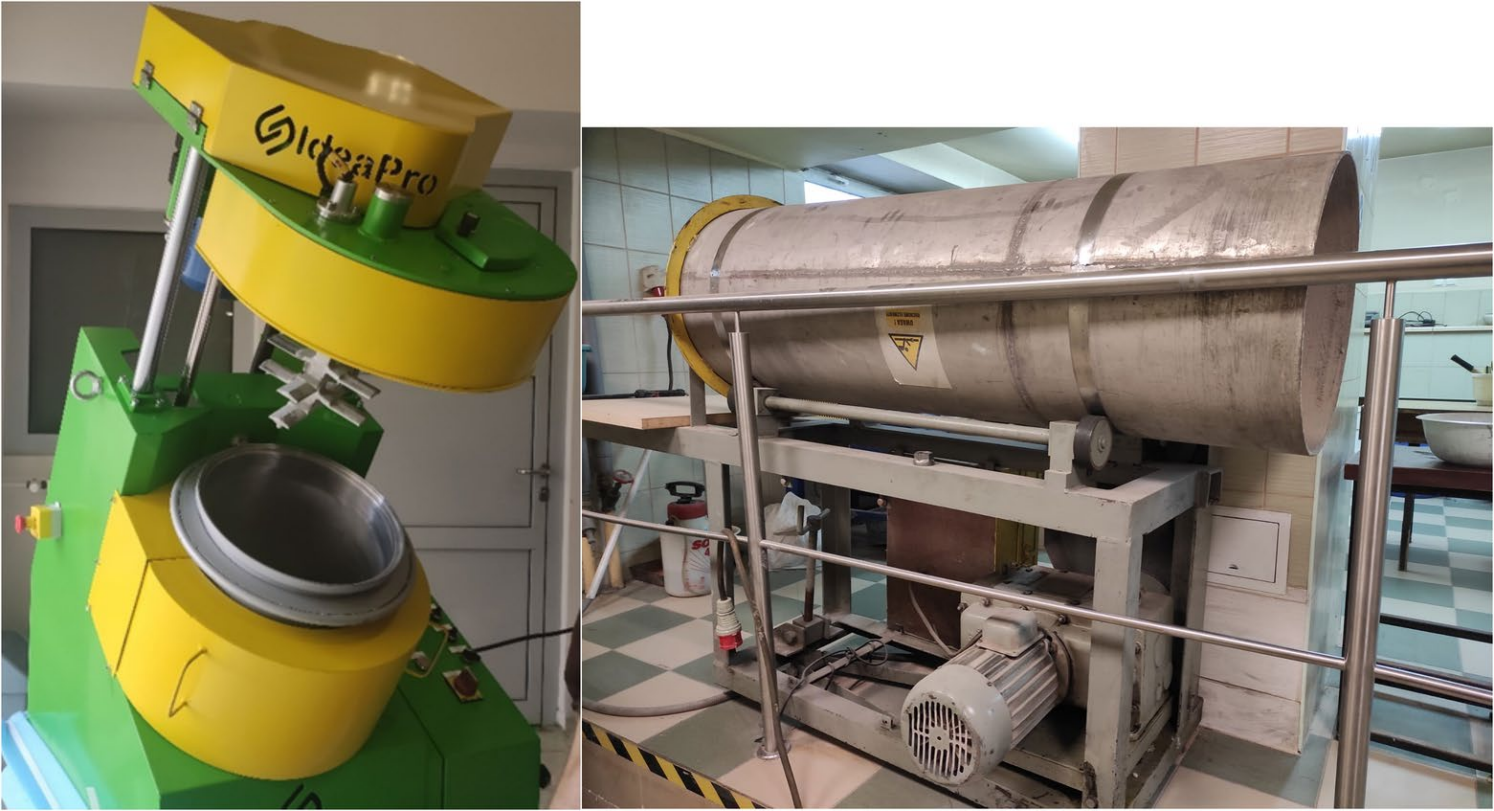
Dynamic counter-rotating granulator manufactured by Idea-Pro (**a**) and drum granulator (**b**), both equipped with the Department of Environmental Engineering at AGH.

Dynamic granulation, takes place in dynamic counter-rotating granulators with variable rotation speed of the drum and mixer with opposite directions of rotation (Fig. 5a). The machine enables the processing of various types of consistency into selected forms of granules. Mixing and granulation takes place in one device and ensures the highest standards in terms of product quality, energy consumption and efficiency per volume unit of the technological device. The raw materials become cohesive in a very short time—several dozen seconds, and because it is a closed chamber system, dust formation is limited to a minimum. Drum granulation—the process is characterized by continuity thanks to the movement of the granulated material inside an open drum inclined at a small angle (Fig. 5b).

**Figure 6:**
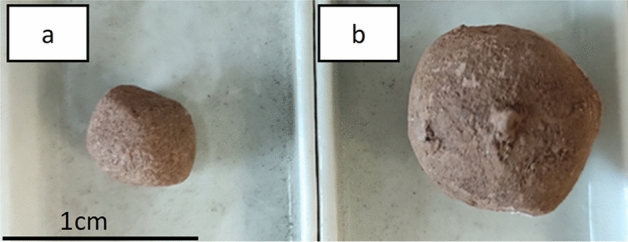
Aggregate granules produced by the dynamic (**a**) and drum (**b**) methods.

Two methods of producing granules were used in the research. Using a dynamic counter-rotating granulator and a drum granulator. In the first case, the granules were smaller (on average 5 mm in diameter, Fig. 6a) and the process occurred much faster, within a few minutes. However, in the second one, a smaller number of balls with a coarser fraction was obtained (approximately 10 mm on average, Fig. 6b). The produced granulates were hardened using a combined method—sintering and hydration.

## Results and discussion

### Formula selection

In order to obtain the best product, three mixtures were created containing different amounts of aggregate ingredients, as shown in Table 3.

**Table 3:** Raw material compositions of the produced aggregates.

Sample acronym	Granulation type	Clay	Concrete dust	PET residues
		%wt		
I GD	Dynamic	40	30	30
II GD		42	42	16
III GD		32	56	12
I GB	Drum	40	30	30
II GB		42	42	16
III GB		32	56	12

### Aggregate sintering process

Sintering is a basic technological process in the production of materials with a fixed shape. When heated to an appropriate temperature, lower than the melting point, the set of contacting grains bond together, forming a polycrystal. The essence of this process are mass transfer mechanisms that lead to macroscopic changes in the material. These include: reduction of porosity and the accompanying thickening and shrinkage of the sintered system, as well as an increase in its mechanical strength. The basic driving force of the sintering process is the energy of the free surfaces of the set of particles. The specific surface area, which is the total surface area of the particles of crushed material per unit mass, is directly proportional to the susceptibility to sintering. In other words, the finer the mineral fraction, the faster the shape preservation process. The tested materials are fine-grained, so the transport of matter will occur quickly and uniformly^39^. Generally, sintering is a three-step process:

- stage I – initial sintering phase – observed when the material temperature is approximately 0.25 of the melting temperature, no shrinkage is observed at this stage, the original arrangement of layers in the aluminosilicates remains intact, the products are dried with a gradual increase in temperature to 200 °C. This process is characterized by the removal of the remaining free water, the content of which in the raw material is still several percent.
- stage II – intermediate phase – occurs when the material temperature is 0.25–0.75 of the melting point, at this stage the beginning of shrinkage is noticeable, grain growth and material thickening occur. During the second firing period, also called the dehydration period, which includes a further increase in temperature to approximately 600°C, chemically bound water is released. At the same time, the dehydration process decomposes organic substances and begins to decompose chemical compounds and minerals.
- stage III – final phase – end of the thickening phase, transformation of open pores into closed ones and their partial disappearance, with continuous grain growth. This stage, called the vitrification period, is characterized by significant changes in the mineralogical composition of the mass.

In the granule sintering process, attempts were made to prevent stage III from fully developing in order not to close the high porosity of the material. The set temperature was intended to roast (chemically change) raw materials undergoing thermal dissociation. As a result, this was to increase the porosity of the aggregates due to escaping gases and burning of organic parts, which also contributes to a decrease in the apparent density and an increase in the absorbability of the aggregate. Changing these properties will have a positive effect, for example, on the water retention of aggregates. This optimally selected temperature ensures the formation of a durable sinter, and durability is achieved through the glassy phase that sticks the powder particles together^39–41^.

**Figure 7:**
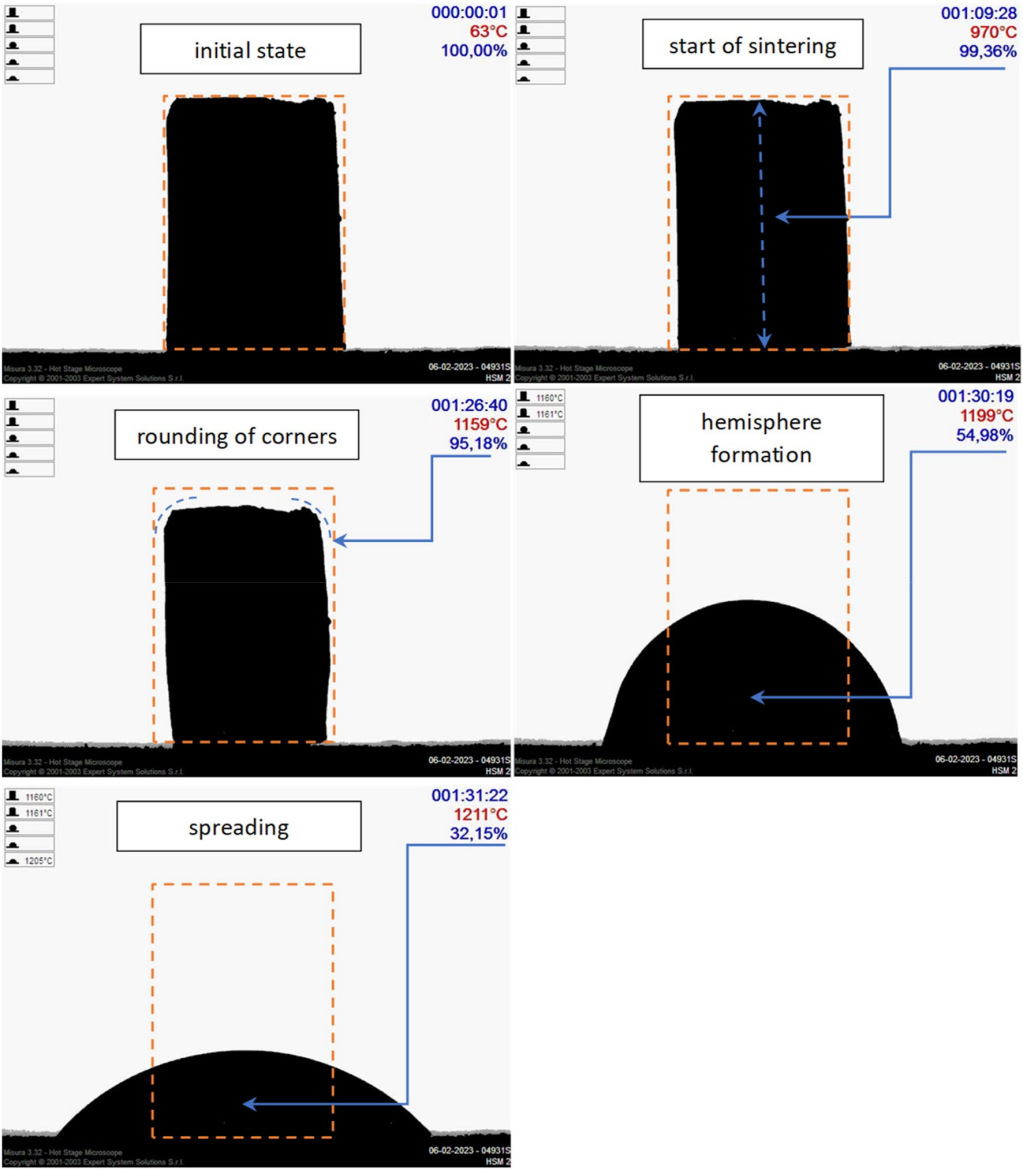
Example images from a high-temperature microscope for a sample with the method of determining characteristic temperatures.

Additionally, Fig. 7 shows an example image obtained from a high-temperature microscope.

The measurement involves observing changes in the sample's contours as the temperature increases, which allows the determination of characteristic temperatures, such as:

- Sintering temperature Ts – temperature at which the sample reaches 99% of its initial height;
- Softening temperature Tm – also referred to as the beginning of melting, at which the sample shows a pronounced rounding of the edges;
- Hemisphere temperature Tp – the height of the sample is half the diameter, it determines the end of its melting;
- Spreading temperature Tr – the height of the sample is equal to 1/3 of the initial height ^42^.

**Figure 8:**
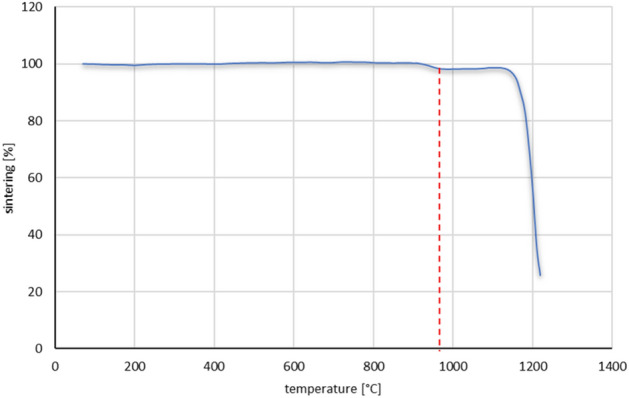
Average sintering diagram of aggregate, the minimum sintering temperature is marked with a red line.

The results from the measurement of characteristic temperatures are presented in Table 4 and Fig. 8. Samples with extreme clay contents were selected for testing, i.e. sample II with a clay content of 42 wt%, and sample III with a lower clay content of 32 wt%, because this raw material is responsible for obtaining the sinter (Table 3). The granulation process method had no effect on the sintering process. No significant differences were noticed in the sintering process.

**Table 4:** Summary of characteristic temperature values [°C].

Sample	GDII, GBII	GDIII, GBIII
Sintering temperature T*s*	970	974
Softening temperature T*m*	1161	1158
Hemisphere temperature T*p*	1201	1198
Spreading temperature T*r*	1213	1215

Based on the obtained test results, it was decided to use firing at a temperature of 1000°C (slightly above the minimum sintering temperature). This temperature guarantees obtaining a durable sinter, and at the same time no significant shrinkage of the sample is observed and the porosity does not close.

### Sintered aggregate

**Figure 9:**
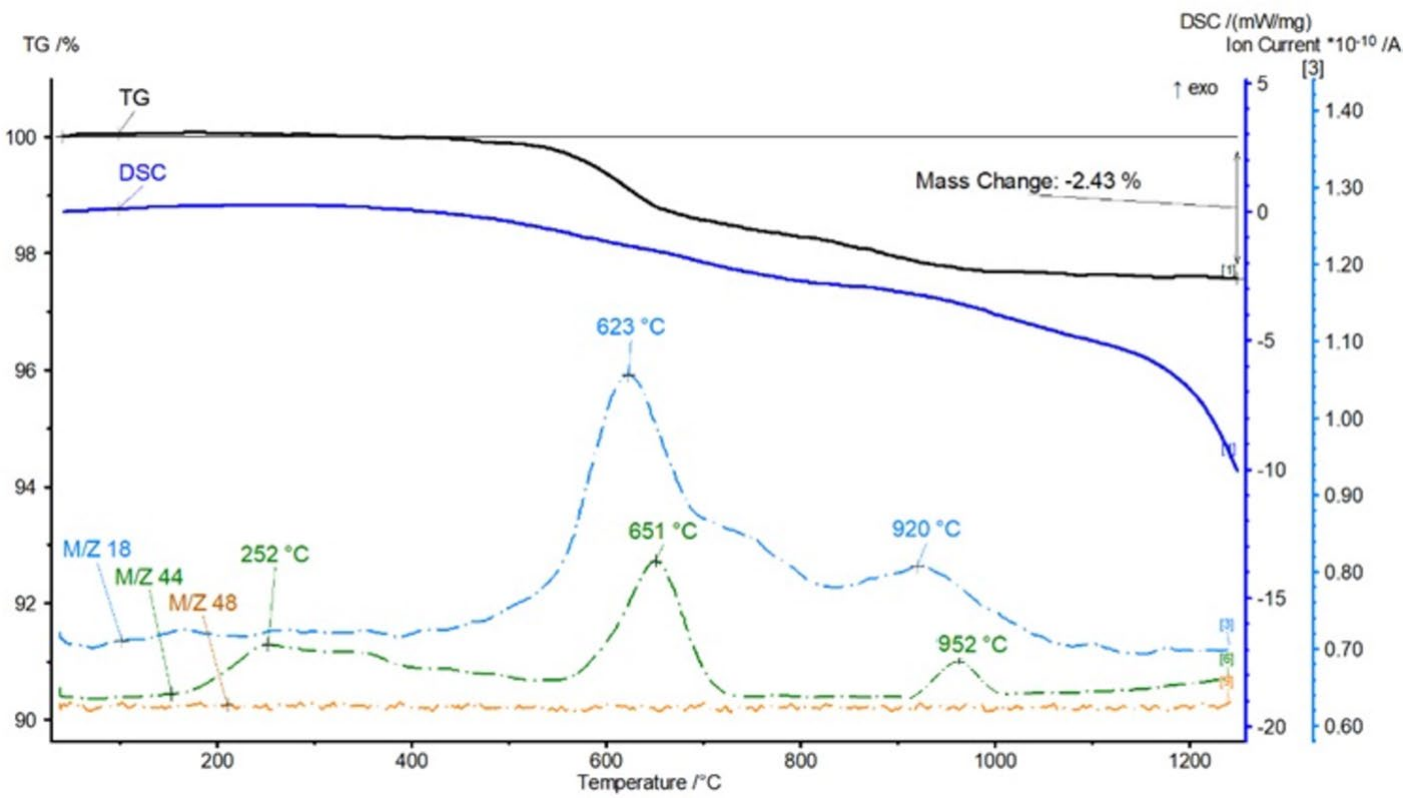
Thermograph of aggregate mass with composition III.

The tested materials have a complex mineralogical composition, hence the sintering system is complicated. The appropriate amount of calcium carbonate in the mass causes the formation of CaO (decarbonation of CaCO_3_), and consequently reduces the viscosity of the liquid phase formed at high temperatures and facilitates the sintering process. The second effect is the formation of pores as a result of decarbonation, which create a network of interconnected channels (open porosity increases). In raw materials with an excess of CaCO_3_, anorthite, calcium aluminates and silicates, and braunmilerite are formed at temperatures above 960 °C (Fig. 9). The decarbonization reaction of calcite and dolomite proceeds intensively at normal pressure. The presence of dehydrated clay and inorganic admixtures Fe_2_O_3_, TiO_2_, SiO_2_ and others contribute to the acceleration of the decarbonization reaction.

The dehydration of clay minerals is primarily influenced by the firing environment. In addition, the presence of Fe^2+^ in clays promotes the formation of new phases. The group of reactions in solid phases of clays that take place through the transport of matter can be described using the following formulas:

3(Al_2_O_3_·2SiO_2_) → 3(3Al_2_O_3_·2SiO_2_) + 4SiO_2_, (mullite, cristobalite).

4(Al_2_O_3_·2SiO_2_) + 3Fe_2_O_3_ → 4(FeO·Al_2_O_3_) + 4(2FeO·SiO_2_) + 7SiO_2_ + 1^1^/_2_O_2_, (hercynite, fayalite, cristobalite).

4(Al_2_O_3_·2SiO_2_) + 6 FeO → 4(FeO·Al_2_O_3_) + 4(2FeO·SiO_2_) + 7SiO_2_, (hercynite, fayalite, cristobalite).

3(Al_2_O_3_·2SiO_2_) + 3CaCO_3_ + 4SiO_2_ → 3(CaO·Al_2_O_3_ 2SiO_2_) + 3CO_2_, (anortite).

Cristobalite formed during firing of montmorillonite-kaolinite raw materials "loosens the body" and increases its permeability and water absorption^39,43–46^.

In order to determine temperature transformations and the mass loss of the tested samples, DSC analyzes were performed. The tests were performed on dried and powdered samples. The measurement results are presented in Fig. 9. When roasting clay minerals, structural changes occur, mainly involving the removal of hydroxyl groups from their structures (dehydroxylation) and, consequently, the formation of active forms. Mass loss is recorded in the temperature range 610–650°C and is caused by dehydroxylation of clay minerals. Dehydroxylation of clay minerals is a complex process that occurs in a wide range of temperatures, even up to 1200°C, where high-temperature swelling is observed. Since the second component was recycled concrete dust, thermal effects will also come from the decomposition of this material. The analyzed thermogram shows a blurred peak starting at a temperature of 250°C up to a temperature of 400°C. In the range of such temperatures, ettringite is completely dehydrated and the dehydration of the C-S–H phase begins. Dehydroxylation of portlandite also partially occurs. It turns into free lime, which allows it to re-bind when in contact with water. At a temperature of 600°C, most of the C-S–H phase decomposes. This makes roasting recycled fines at a temperature level of 650°C a favorable phenomenon. This is related to the binding properties of this fraction and the possibility of using it as a substitute for cement or an active additive to the composite. Weight loss is also observed around 900–1000°C, so it can be concluded that the samples decompose carbonates and the remains of clay minerals.

#### Leaching of soluble compounds and secondary hydration

The hydration process is a multi-stage process. However, it seems that from the point of view of using calcined concrete dust and PET ash as a grout ingredient, the most important thing is the hydration of tricalcium aluminate. This hydration is complicated, and the product of this reaction are hydrated calcium silicates insoluble in water^46^. To ensure a fully developed hydration process, the samples were maintained in distilled water for 28 days. Samples of fired aggregate with cement dust were subjected to an additional hydration process. The hydration mechanism of calcium aluminates consists of the dissolution process, where the anhydrous phases of clay cement dissolve and then precipitate from the solution in the form of hydrates (chemical compounds from the CaO·Al_2_O_3_·H_2_O system). There are three main phases of the hydration process:

- Dissolving,
- Nucleation,
- Precipitation.

**Figure 10:**
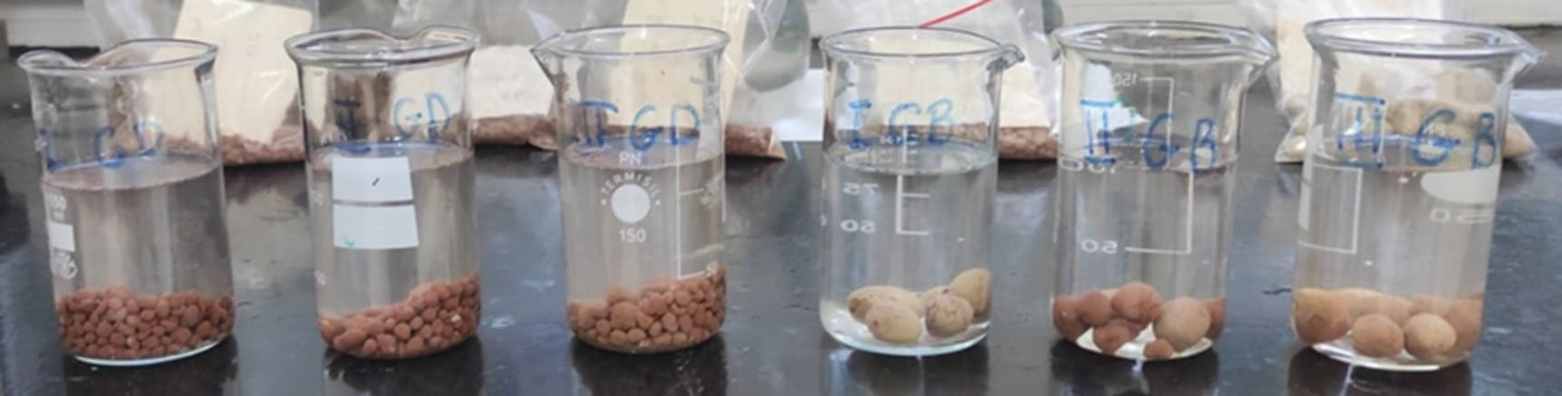
Samples tested for leachability.

The hydration process is initiated by hydroxylation of the cement surface. In the next stage, the cement dissolves in water and releases calcium and aluminum ions into the solution. When the ion concentration exceeds the hydrate solubility level, a small amount of hydrate gel is formed. Dissolution continues with a simultaneous increase in the concentration of calcium and aluminum ions in the water until the saturation level is reached. Crystal nuclei are then formed in large numbers—the nucleation phase. Hydrates begin to precipitate, which leads to a decrease in ion concentration. This is a dynamic process that leads to the dissolution of the rest of the anhydrous cement. In the physical sense, we are dealing with the growth of hydrated crystals that interlock and bond with each other, resulting in the formation of a monolith on a macro scale. The driving force is the lower solubility of hydrates in water than that of anhydrous calcium aluminate. Hydration is a process involving the transfer of ions into solution. This can be confirmed using conductometric measurements. For this purpose, a cement sample is placed in water and tested for ionic conductivity. Its value increases as the number of ions per unit volume increases. For this purpose, tests of the concentrations of substances soluble in water were carried out by performing electrolytic conductivity tests. Additionally, pH changes were monitored. For this purpose, fired aggregate with an appropriate weight of approximately 10 g was immersed in 100 ml of distilled water (Fig. 10). Table 5 shows the measurement results. The parameters of distilled water used for testing were pH 6.04 and conductivity 4.25 µS. Changes in aggregate mass were also recorded after 14 and 28 days, thus checking the condition of the sinter and its resistance to water.

**Table 5:** Results of measurements of changes in pH and conductivity of solutions.

Time	Parameter	I GD	II GD	III GD	I GB	II GB	III GB
Day 1	pH [-]	11.00	11.21	11.78	11.03	11.59	11.67
Day 2		10.40	11.00	11.80	10.40	11.67	11.40
Day 3		9.80	10.49	11.16	10.35	11.40	11.41
Day 7		9.87	10.41	10.98	10.00	10.91	11.03
Day 14		9.65	10.30	10.36	9.16	9.64	9.72
Day 28		9.34	9.87	9.90	9.16	9.56	9.60
Day 1	Conductivity [µS]	107	175	250	189	184	232
Day 2		464	482	544	442	431	525
Day 3		691	723	757	703	699	742
Day 7		456	480	452	432	460	566
Day 14		250	275	237	189	184	192
Day 28		258	265	243	201	164	132
Day 1	Dry weight [g]	10.6567	10.0892	10.7055	10.0908	10.3104	10.4344
	Saturated weight [g]	14.8484	15.5041	15.7730	15.5412	15.1895	14.4545
Day 14		15.1304	15.6010	16.1869	15.5705	15.2077	15.0797
Day 28		15.2081	15.6103	16.4311	15.6730	15.2927	15.3022

There are three stages that can occur in an ionic conductivity test:

1. Rapid increase in conductivity, associated with a sharp increase in the amount of Ca^2+^ and [Al(OH)_4_]^-^ ions. During this process, the slow deposition of primary hydrates in the form of a gel is visible.
2. Saturation state—where crystal nuclei are formed
3. Crystallization of hydration products^47–49^

**Figure 11:**
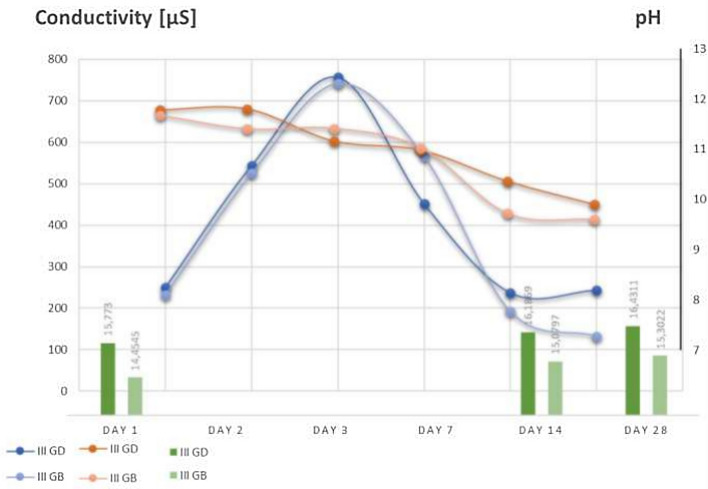
Variability of conductivity and pH and mass increase over time.

The test results confirm the ongoing hydration process. After mixing the aggregate with water, the liquid phase is rapidly saturated with calcium ions, and the pH of the solution increases rapidly. On the first day of measurement, the highest pH values are observed in the case of aggregates with the highest amount of cement (III GD and III GB; pH above 11. The high pH persists for at least 2 weeks and in the following days it begins to slowly decrease, but does not reach neutral values. At the same time, with changes in pH, changes in the conductivity of the solution are observed. In the dissolution phase, it is the highest (reaching values above 750 µS in the case of aggregate samples with the highest cement content (Fig. 11). About 24 h after the start of the measurements, the conductivity begins to decrease because crystallizing phases begin to precipitate aluminate, while K + , Na + and OH- ions remain in the solution. Hence the persistently high pH. Crystallization of cement hydration products causes an increase in the mass of the granules (Table 6). The hydration process also causes additional hardening of the aggregate.

**Table 6:** Measurements of changes in pH and conductivity of solutions—second measurement series.

Time	Parameter	I GD	II GD	III GD	I GB	II GB	III GB
Day 1	pH [-]	7.23	7.12	7.54	7.29	7.70	7.56
Day 2		7.17	7.28	7.49	7.26	7.48	7.79
Day 7		7.27	7.18	7.31	7.16	7.32	7.61
Day 14		7.02	7.20	7.34	7.08	7.34	7.50
Day 1	Conductivity [µS]	234	219	261	214	221	241
Day 2		297	278	290	271	256	289
Day 7		279	281	250	250	242	249
Day 14		283	275	267	229	256	271

After 28 days, a second test was carried out in water, and pH and conductivity were similarly measured. For this purpose, aggregate samples were filtered and poured with fresh distilled water with a pH of 6.17 and a conductivity of 6.13 µS. Table 6 shows the measurement results. The pH indicator is neutral throughout the entire research period, and no increased salt leachability was observed.

#### Bulk and apparent density

All obtained granulates had an apparent density below 2g/cm^3^, which meets the standards for classifying the aggregate as light. Slightly lower bulk densities were observed in materials obtained using the drum method—this is due to the coarser and less differentiated aggregate fraction. The lowest densities were obtained in samples of composition III containing 52% of concrete dust, however, these values may change due to the ongoing hydration process. It should be noted, however, that the deviations are small and may be negligible on a larger scale. Table 7 shows the measurement results.

**Table 7:** Average apparent and bulk densities of the tested samples.

Parameter	I GD	II GD	III GD	I GB	II GB	III GB
Bulk desity [g/cm^3^]	0.79	0.80	0.76	0.65	0.70	0.68
Apparent density [g/cm^3^]	1.43	1.43	1.31	1.39	1.35	1.23

#### Kinetics of water release

**Figure 12:**
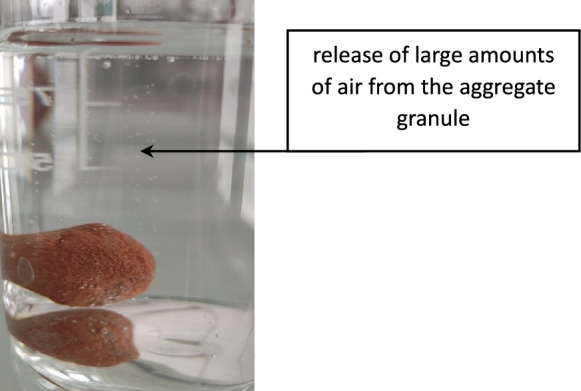
Air escaping from the aggregate granules.

The obtained aggregates are very porous, it is open porosity. Figure 12 shows the dynamics of water absorption.

**Figure 13:**
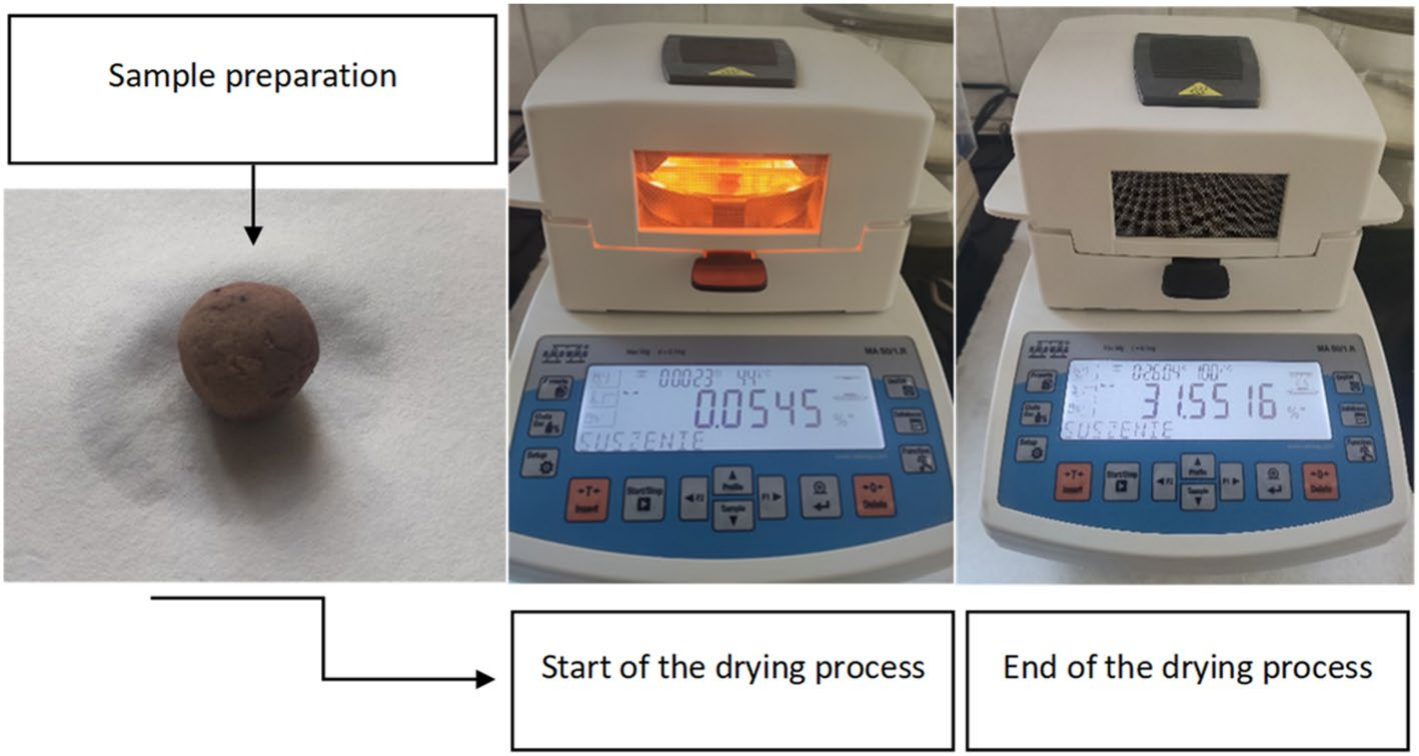
Measurement of drying speed, preparation of granules for testing (**a**) beginning of drying (**b**) recorded weight loss after drying (**c**).

Since the aggregates are intended for use on green roofs, tests were carried out using a precise moisture analyzer with a drying chamber (Fig. 13), which ensures a uniform drying temperature during measurement. Measurements were carried out at a temperature of 100°C, the average initial weight of the samples was 1.5 g. Tables 8 and 9 present the measurement results.

**Table 8:** Recorded water discharge data.

Time, [min]	I GB	II GB	III GB	I GD	II GD	III GD
	Loss of weight [%wt]					
5	4.3	4.2	4.8	9.88	10.17	10.9
10	11.25	9.2	11.6	19.76	22.12	21.99
15	19.21	15.59	18.35	26.99	27.11	28.76
20	25.95	20.92	24.9	31.79	30.28	33.59
25	29.78	25.85	31.08	34.29	38.07	43.24
30	32.84	29.39	34.91	–	–	–

**Table 9:** Total weight loss depending on time.

	I GB	II GB	III GB	I GD	II GD	III GD
total weight loss [%wt]	32.86	33.83	35.89	34.45	39.02	45.62
total water release time	30 min 41 s	35 min 18 s	34 min 4 s	25min23	25 min 31 s	23 min 59 s

It turns out that the granulation method affects the rate of water release—granules produced with a drum granulator release water much longer (Table 8), on average the difference is 8 min. This is influenced by both the aggregate fraction and the type and size of porosity. Materials with open porosity will behave differently from those with partially closed porosity. The more open pores, the greater possible water retention. The composition of the samples also influenced the drying dynamics. Interestingly, samples with composition I absorbed the least amount of water, which is justified because they contained the largest amount of clay, which partially forms a glassy phase during sintering, closing the pores.

#### Strength tests on granules

The lightweight aggregates usually do not carry heavy loads, so there is no need for them to have high crushing properties. However, they should be characterized by their abrasion resistance. which is a advantages of transport. If such aggregate is designed for a green roof it should not form a dusty fraction during the operation of the green roof, which settles at the bottom of the layer and can interfere with the drainage of excess water. Table 10 shows the results of the crushing and abrasion strength measurements. Measurements were taken on the same samples before and after the hydration process. Each sample was tested five times, and the table shows the arithmetic mean drawn from the measurements.

**Table 10:** Crushing strength and abrasion resistance.

Parameter		I GB	II GB	III GB	I GD	II GD	III GD
Crushing strenght [N]	Before hydration	166.6	210.9	161.4	105.0	120.2	106.7
	After hydration	199.4	247.1	284.3	162.8	159.6	178.3
Strength gain [%]		19.8	17.1	76.3	54.2	32.5	67.9
Abrasieveness [%]	Before hydration	3.48	4.27	3.83	5.18	3.49	2.90
	After hydration	3.21	3.56	2.79	4.23	3.12	3.03

The results of the crushing tests clearly show that the hydration process strengthens the granules. In each test, an increase in crushing strength was observed after 28 days of care in water. This increase ranged from approximately 17 to more than 75 percent. The highest strengths of approx. 280 N were observed in the samples granulated using the drum method where the proportion of concrete dust was highest (sample composition III, Table 3). As far as the granulation process is concerned, the drum method produces more packed and stronger granules. The abrasiveness is similar in all cases and amounts to a few percent.

#### SEM scanning microscopy,

**Figure 14:**
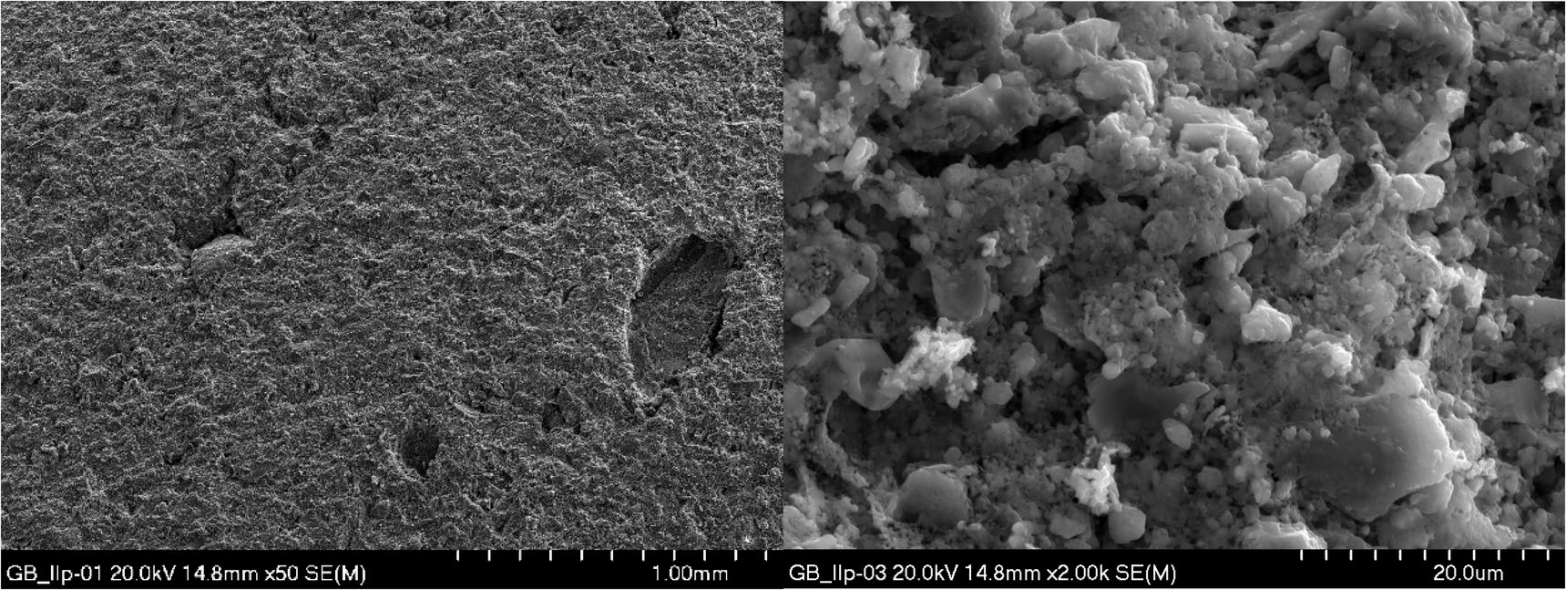
Microphotograph of the sample GB II surface—magnification 50 × and 2000 ×, respectively.

**Figure 15:**
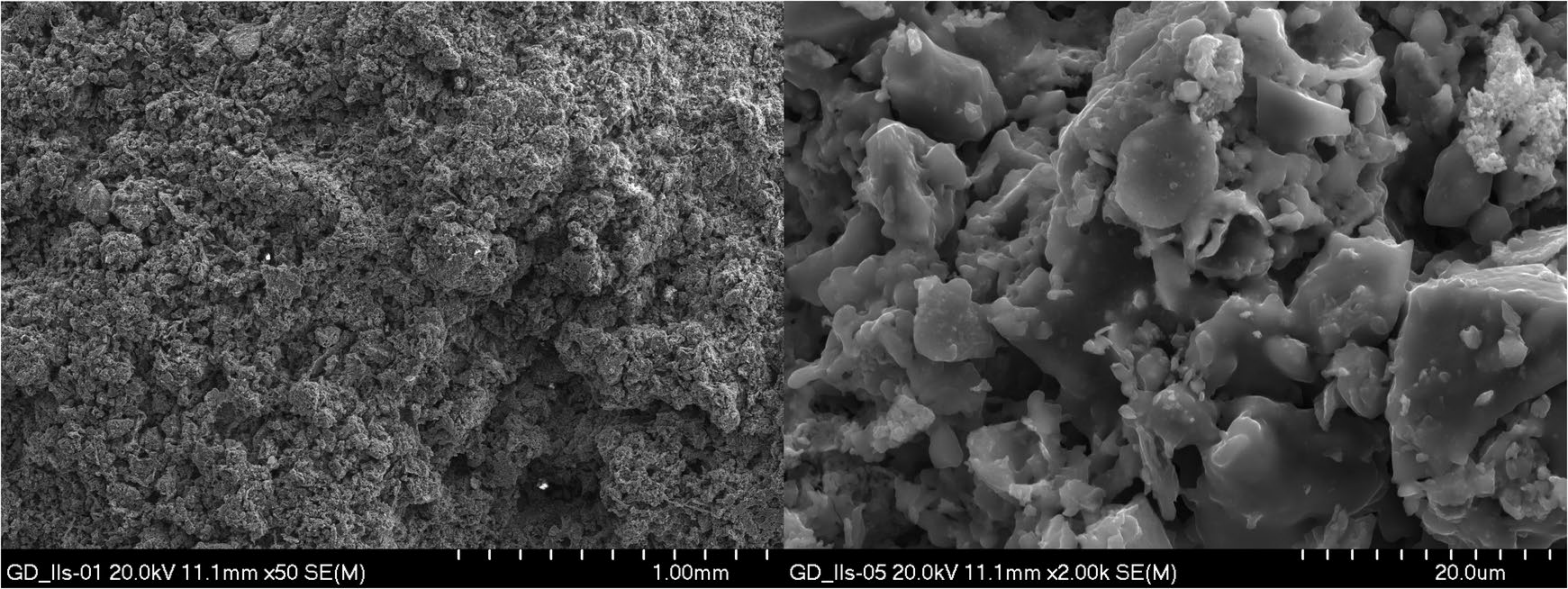
Microphotograph of the GB II sample fracture—magnification 50 × and 2000 ×, respectively.

**Figure 16:**
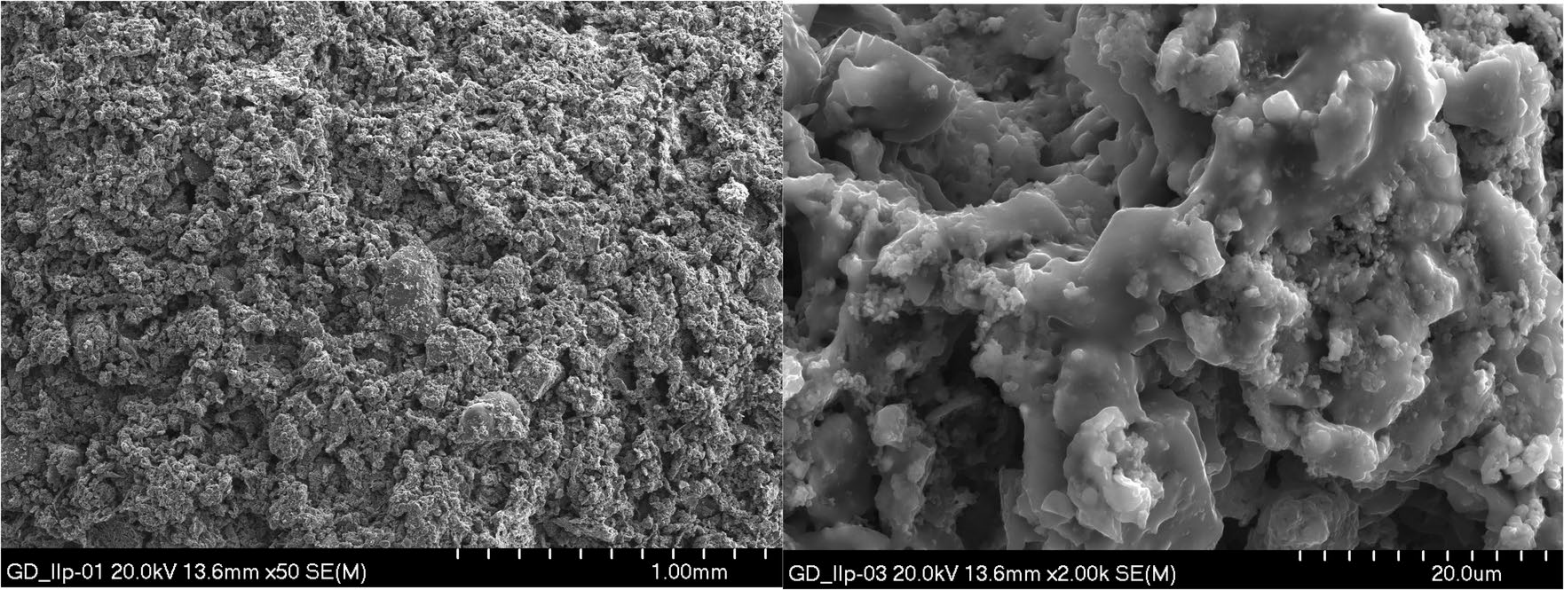
Microphotograph of the GD II sample surface—magnification 50 × and 2000 ×, respectively.

**Figure 17:**
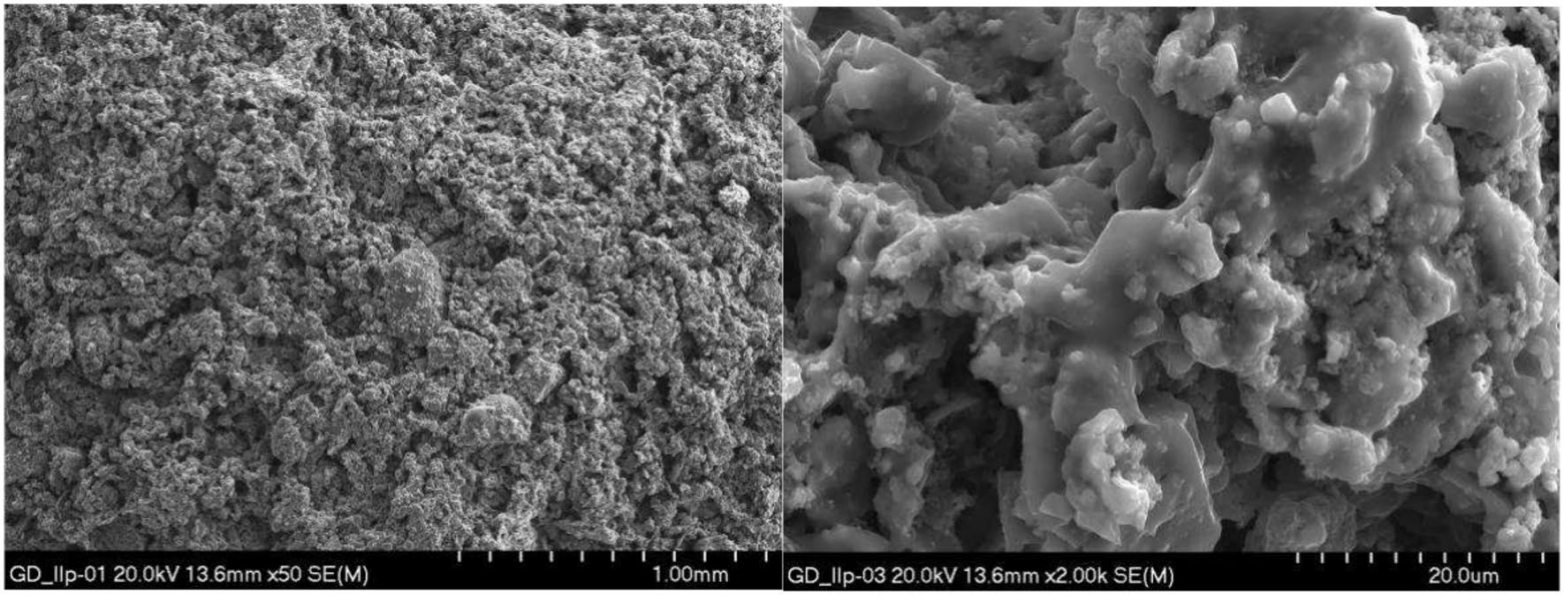
Microphotograph of the GD II sample fracture—magnification 50 × and 2000 ×, respectively.

Microstructure tests of selected aggregates were carried out. Samples of composition II produced by both granulation methods, i.e. drum and dynamic, were selected for testing. The surface and interior of the samples were examined. EDS tests of the materials were also performed. Microscopic photos are shown in Figs. 14, 15, 16, 17. Porosity is higher in aggregate formed using the dynamic granulation method, the structure is more porous, the grains are less stuck together, this is especially visible at lower magnifications of 50 times – Figs. 16 and 17. The aim of the research was to produce a material with high porosity that can absorb large amounts of water . Both granulation methods generally meet these assumptions, however, the microstructure of the GD granules seems to be more favorable.

**Figure 18:**
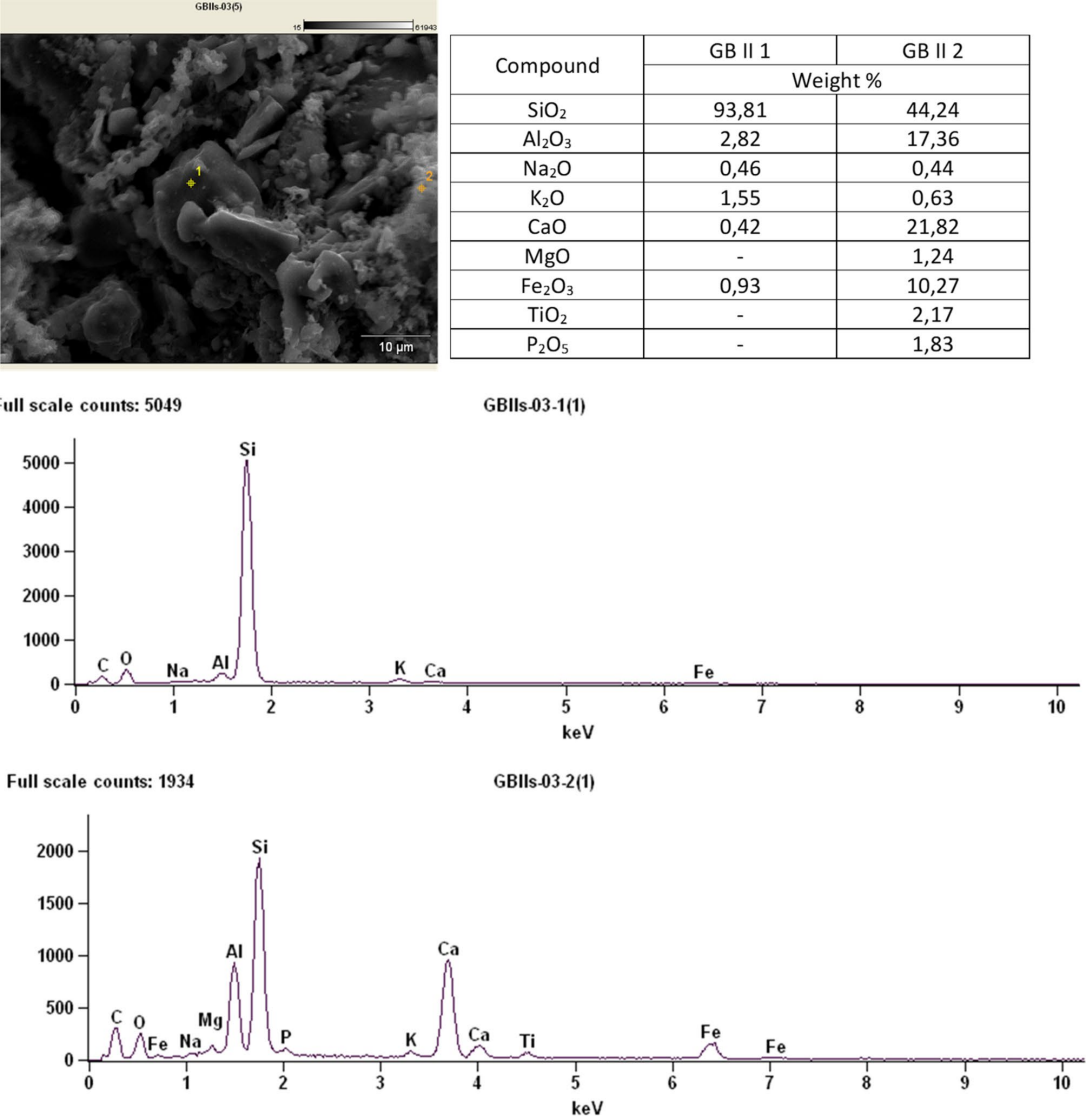
Microphotograph with marked EDS analysis points and the results of this analysis.

The GB II sample was selected for testing its chemical composition using EDS energy dispersive spectroscopy—there is no need to test the material made with the second method because the material components and their quantity are the same. Elemental analysis at various points shows that the chemical composition of aggregates is diverse, there are places where there is mainly silica up to 94 wt%, but there are also micro-areas with very different contents of the main oxides, i.e. SiO_2_—44 wt%, CaO—22 wt% Al_2_O3—17%tue An example analysis is shown in Fig. 18.

## Conclusions

The problem of disposal and management of solid waste materials has become one of the main environmental, economic and social problems. The use of solid waste in the production of lightweight aggregate not only solves the problem of waste disposal, but also helps in transforming waste into useful and profitable products.

Recycling PET waste into lightweight aggregates seems to be a feasible solution, not only to the problem of disposal of this type of waste, but also an economical option for the production of lightweight aggregates. Concrete dust from demolition of difficult use is used in this technology as a material that limits plasticity, but also supports hardening through secondary hydration.

The aim of the research was to produce a light aggregate from by-products and waste materials, this goal was achieved, a light and highly porous aggregate was obtained that can be used in various industries, including sustainable architecture.

In further research, the authors will use other plastic raw materials, e.g. clays produced during the washing and processing of gravel, which are so far treated as waste material and stored in water reservoirs.

We are currently conducting research in the use of these aggregates as substrates for green roofs. We believe that this direction is the most promising. Classic expanded lightweight aggregates, such as LECA, do not have the necessary open porosity and, despite their advantages, are less able to retain the water available to plants.

## Data Availability

The datasets used and/or analyzed during the current study available from the corresponding author on reasonable request.
